# iACT-CEL: A Feasibility Trial of a Face-to-Face and Internet-Based Acceptance and Commitment Therapy Intervention for Chronic Pain in Singapore

**DOI:** 10.1155/2017/6916915

**Published:** 2017-02-23

**Authors:** Su-Yin Yang, Rona Moss-Morris, Lance M. McCracken

**Affiliations:** ^1^King's College London, Health Psychology Section, Psychology Department, Institute of Psychiatry, Psychology and Neuroscience, London SE1 9RT, UK; ^2^Pain Management Clinic, Tan Tock Seng Hospital, Singapore 308433

## Abstract

Psychological interventions are increasingly utilising online or mobile phone based platforms to deliver treatment, including that for people with chronic pain. The aims of this study were to develop an adapted form of Acceptance and Commitment Therapy (ACT) for chronic pain in Singapore and to test the feasibility of elements of this treatment delivered via the internet and email.* Methods*. Thirty-three participants recruited from a tertiary pain management clinic and via the clinic website participated in this program over a period of five weeks with a 3-month follow-up. Treatment outcomes were assessed at three assessment time points.* Results*. 90.9% of participants completed the program, with 81.8% reporting high treatment satisfaction. Significant changes in depression, *t* = 3.08, *p* = 0.002 (baseline to posttreatment), *t* = 3.28, *p* = 0.001 (baseline to follow-up), and pain intensity, *t* = 2.15, *p* = 0.03 (baseline to follow-up) were found. Mainly small effect sizes (*d* = 0.09–0.39) with a moderate effect size (*d* = 0.51) for depression were found at posttreatment. Clinically meaningful improvement in at least one outcome was demonstrated in 75.8% of participants.* Conclusions*. An adaptation of ACT for people with chronic pain in Singapore appears promising. Optimal treatment design and more effective ways to target outcomes and processes measured here are required.

## 1. Introduction

Studies around the world reflect a consistently high prevalence of chronic pain [1–3], and it is both a large and growing world health priority. The complexities of managing chronic pain [4, 5], inadequate healthcare resources [6], limited accessibility to psychological treatments [7], and high treatment costs [8, 9] appear to confound an adequate global response to pain.

Online or mobile phone based treatments for chronic pain have the potential to overcome access barriers such as cost [10], competing time commitments, and transportation challenges and to address issues like perceived stigma associated with psychological treatment [11]. Such treatments may increase the total health impact with the same amount of therapist time [12].

The most common psychological approach to chronic pain is Cognitive-Behavioral Therapy (CBT) [13]. Traditionally CBT-based interventions are aimed at modifying patients' unhelpful beliefs about pain. Within the CBT model, it is proposed that unhelpful beliefs about pain contribute to a perpetuation of the pain experience. An ability to modify these unhelpful beliefs would in turn modify maladaptive pain-related behaviors and subsequently improve the overall pain experience [14]. More recently, contextual cognitive behavioral approaches, including Acceptance and Commitment Therapy (ACT), which include acceptance and mindfulness approaches as key examples, are part of what is sometimes called the “third wave” of psychological treatments. Psychological treatments within the “third wave” include a shift in philosophical assumptions, theory, and methods from previous treatments [15]. However, like traditional CBT, ACT similarly aims for cognitive process change and improved behavioral outcomes [16] and adopts methods used in other established therapies to achieve this aim. Methods in ACT include mindfulness, exposure, behavioral activation, and skills training as examples [17]. The main difference between traditional CBT and ACT is that while the former aims for change in the content of thoughts, beliefs, and feelings, ACT aims more for changes in the influences exerted by these experiences on behavior. ACT is also in general more focused on performance enhancement and less focused on symptom reduction.

The conceptual framework behind ACT is the psychological flexibility (PF) model [17]. PF relates to one's capacity to remain in the present moment; to be conscious of thoughts, feelings, and potentially undesirable internal experiences; and to persist or change behavior aligned with chosen values. PF includes a set of six core component processes, which are “acceptance,” a willingness to have unwanted experiences including difficult emotions and pain [17]; “cognitive defusion,” the process of seeing thoughts as they are and separate from the events to which they relate; “present moment awareness,” a focus on the present experience rather than situations in the past or those that will occur in the future; “self-as-context,” a kind of perspective taking that includes a distinction between psychological experiences and the person who has them; “values,” general life directions that function as guiding principles in one's life; and “committed action,” behavior patterns consistently aligned with one's goals and values [17]. These processes of PF have also been conceptualized as behavior that is “open (acceptance-cognitive defusion), aware (in the present moment-self-as-context), and engaged (values-committed action)” [18].

Results from treatment outcome studies across diverse clinical settings and conditions have broadly demonstrated consistent relations between measured processes of PF and patient functioning, including both physical and emotional functioning [19]. These results particularly include measures of acceptance, values-based action, and present moment awareness taking a dominant focus [20–22]. In recent years, measures of cognitive defusion [23] and committed action [24] have been developed and validated in the chronic pain population as well. Results from these studies broadly support the utility and generalizability of the ACT model and related measures in chronic pain.

While the literature on ACT and PF in general is growing, there are limited data on the relevance, applicability, and acceptability of these approaches in non-Western populations [25]. ACT is thought to be well suited for people from diverse cultures and populations. ACT's emphasis and focus on the individual's goals and values crafted within the context the individual brings to treatment, and the use of personally relevant metaphors in treatment can contribute to a culturally sensitive approach within ACT. The ACT in treatment can thus help guide the type of treatment adaptations needed to more effectively tailor treatment to diverse populations [26].

Internet-delivered ACT-based interventions for chronic pain have demonstrated significant reductions for pain related distress, anxiety and depression [27], pain interference, disability, and catastrophizing [28], at 6-month follow-up in the ACT intervention. Though not demonstrated as superior to CBT, ACT has been recognized as a legitimate treatment alternative for people with chronic pain [29].

The use of computer and communications technology as part of treatment delivery has been suggested in previous work as a means to increase psychological treatment uptake for chronic pain in Singapore [7, 30]. Statistics provided by the Infocomm Development Authority of Singapore (IDA) places Singapore as a technologically savvy country with 88% of households having access to the internet [31]. As such, tailoring an internet-based ACT treatment for chronic pain, designing it in a form that is culturally sensitive, and testing this approach as part of a feasibility trial in Singapore appear worthwhile.

This study aimed to develop an adaptation of an ACT-based treatment that is suitable for people with chronic pain in Singapore and to test the feasibility of the program delivered partly through an internet-based platform [32]. Assessment here included recruitment, retention, treatment expectations, acceptability and satisfaction, and standard clinical outcomes of pain interference, satisfaction with life, pain intensity, depression, and impact of depression. We predicted that we would reach the required recruitment target (*N* = 30) within a 3-month recruitment period and that the majority of participants would complete the modules and assessments and report satisfaction with the experience. Although the trial was not powered to detect significant effects on outcomes, observing potentially clinically meaningful changes in outcomes for a majority of participants was expected.

## 2. Materials and Methods

This study was approved by the Domain Specific Review Board (DSRB: 2014/00641), the local ethics committee in Singapore. All participants provided informed consent to participate in this study.

### 2.1. Design

This was an uncontrolled pre-post study design. Treatment outcomes were measured online via self-report instruments at three time points: (a) baseline, (b) immediately after treatment, and (c) at 3-month follow-up.

### 2.2. iACT-CEL

While the use of Randomized Controlled Trial (RCT) designs for internet-based trials was recommended in a recent Cochrane review [33], this was not done here for several reasons. The primary focus here was feasibility questions. Also, resource and ethical considerations placed restrictions on what could be done. The pre-post design meant that greater attention could be afforded to treatment design and delivery, consistent with preferences observed in previous research in the same setting [34]. Thus, iACT-CEL was designed as a combination of a face-to-face and internet-delivered intervention.

### 2.3. Participants

Participants were recruited from the pain management clinic (PMC) at a tertiary hospital in Singapore and via the PMC website. Participants were included if they were (a) above the age of 21 years old, (b) diagnosed with chronic noncancer pain for more than 3 months, (c) competent in English, (d) able to access and use the internet and e-mail, (e) no current participation or previous participation in a structured approach to CBT for chronic pain in the last 1 year, and (f) their primary doctor's approval to take part in the study.

Participants were excluded if they (a) were diagnosed with a cognitive impairment as documented in neurological or neuropsychological assessment findings and recorded in their medical records, (b) were diagnosed with mental illness or health problems expected to significantly interfere with study participation, or (c) were currently pregnant or breast-feeding. Women who are pregnant or breast-feeding are thought to have another dominating focus (maternal, child, and family) in their life apart from managing pain, at least for the short-term. There is a potential risk that physiological complexities and diverging interests towards pregnancy and related issues may influence their motivation to engage fully in the study. Hence, likely benefits from study participation may not be optimal.

All participants recruited at the PMC were first screened by their attending primary health professional (pain physician, pain nurse, physiotherapist, and occupational therapist or psychologist) immediately after their consult at the pain clinic for eligibility to participate. Participants were screened using a predesigned checklist as well as reference made to their individual medical records to ensure that inclusion criteria were met. Participants who were recruited via the PMC website were firstly screened for eligibility by a trained psychology intern at the PMC, with supervision from a senior psychologist who provided guidance where needed. All participants who provided informed consent had their primary pain physician's permission to participate.

### 2.4. Intervention

The therapist who conducted the intervention held a masters level health psychology degree with 10 years of experience providing treatment for people with chronic pain. She received fortnightly supervision from an experienced senior clinical psychologist.

Participants completed a total of two face-to-face and six online sessions over a period of 5 weeks.  Table 1 provides a schematic overview of the intervention (the full protocol is available from the 1st author). The six online sessions were designed to enhance the primary “open, aware, and engaged” processes of ACT, including acceptance and defusion, present moment awareness and self-as-context, and values and committed action, respectively [18]. A total of seven mindfulness exercises delivered in audio mode were also incorporated into the treatment program as a supplemental focus. Five of these formed a series of optional exercises and the remaining “observing the breath” and “the observing self” exercise incorporated into the main program. Overall, each session included a mix of online “written” exercises, experiential exercises, and metaphors in the form of audio, video, and text. A minimum time of 45 minutes was needed to complete a session in one sitting, similar to time spent in a face-to-face session. No limits were set on the number of times or length of time that participants could access each session.

All communication within the program was handled within a secure encrypted system. Participant numbers were used in all communication. A user database was created to store participants' last logged in information. E-mail interactions initiated by the therapist followed a structured response that included (a) encouragement of participants' progress and motivation to continue with the intervention, (b) clarification of unclear aspects of the intervention, and (c) answering participants' questions. The therapist also responded to separate queries from participants made via e-mail within 24 hrs of receipt. An alternative form of backup communication (phone contact) intended to address technical related issues on the program was provided. Participants were instructed to call for this purpose only after e-mail communication did not resolve the issue at hand.

#### 2.4.1. Cultural Adaptation of ACT Methods for iACT-CEL

A number of cultural adaptations were applied to modify ACT methods to the participant population. A modification of language and the use of culture specific examples formed the main modifications of ACT methods for iACT-CEL. So although the treatment was delivered in English, sentence structure, choice of words, and examples used to illustrate an ACT process, voice accent, and rhythm carried a Singaporean quality. The therapist delivering treatment is Singaporean Chinese and the associated communication style is immediately evident in the delivery of video content used on the program.

### 2.5. Procedure

Participants continued with treatment as usual including medical visits and physiotherapy treatments but did not seek other psychology related treatments while on the program.  Table 2 summarises the study schedule.

### 2.6. Measures

General demographic information was measured at baseline only.

#### 2.6.1. Healthcare Usage

Healthcare use was assessed with a 4-item measure of pain-related medical visits over the past 3 months, including number of doctors seen, number of doctor visits, visits to the accident and emergency care (A & E), and number of days hospitalized.

#### 2.6.2. Survey on Treatment Expectations, Program Acceptability, and Satisfaction

Treatment expectations (see Appendix A) and program acceptability and treatment satisfaction (see Appendix B) were measured by single items that were not part of a validated scale. Items measuring treatment expectations and program acceptability were adapted from Borkovec and Nau's [35] treatment credibility and expectancy questionnaire.

#### 2.6.3. Primary Outcomes

*(1) Brief Pain Inventory (BPI): Interference Scale*. The BPI [36] interference scale is a measure of pain interference on seven daily activities. Participants rate each item on a scale from “0,” “never interferes,” to “10,” “completely interferes.” A single pain interference score is produced from averaging the seven interference items. Adequate internal consistency with Cronbach's alpha ranging between 0.93 and 0.95 has been demonstrated for this scale [37].

*(2) Satisfaction with Life Scale (SWLS)*. This is a 7-item measure of global life satisfaction. The scale has adequate internal consistency (*α* = 0.87) and a test-retest reliability correlation coefficient of *r* = 0.82 [38].

#### 2.6.4. Secondary Outcomes

*(1) Pain Intensity*. An average of the ratings on present and average pain intensity over the past week which was assessed using a “0,” “no pain,” to “10,” “worst possible pain,” numerical rating scale provided an overall pain intensity score [39].

*(2) Patient Health Questionnaire-9 (PHQ-9)*. The PHQ-9 is a ten-item measure of symptoms of depression [40]. The items are scored from “0,” “not at all,” to “3,” “nearly every day.” An index of the severity of depression is obtained from the sum of the first nine items.

The tenth item is a single item measure of the impact of depression. This item is intended and used as a separate index for the measure of the interference and impact of depressive symptoms in one's life [24, 34], specifically in areas related to work, home, and social activities. Participants rate this item as “not difficult at all,” “somewhat difficult,” “very difficult,” or “extremely difficult.” The PHQ-9 has both good internal reliability (*α* = 0.89) and test-retest reliability.

#### 2.6.5. Measures of PF

Measures of PF were included to determine any changes on these potential therapeutic mechanisms.

*(1) Chronic Pain Acceptance Questionnaire-8 (CPAQ-8)*. The CPAQ-8 [41] is an 8-item version of the original 20-item measure of pain acceptance [42]. Participants rate each statement on a scale from “0,” “never true,” to “6,” “always true.” Good internal consistency reliability (*α* = 0.77 to 0.89) has been demonstrated [41].

*(2) Acceptance and Action Questionnaire-II (AAQ-II)*. The AAQ-II [43] is a 7-item version of the original AAQ [44]. It is a measure of general/psychological acceptance. Patients rate each statement on a scale from “1,” “never true,” to “7,” “always true.” The AAQ-II has adequate internal consistency (*α* = 0.78 to 0.88) and a test-retest reliability (*r* = 0.79 to 0.81).

*(3) Committed Action Questionnaire (CAQ)*. The CAQ is an 18-item measure of committed action as defined within the PF model [24]. Patients rate each statement on a scale from “0,” “never true,” to “6,” “always true.” The internal consistency (*α* = 0.87) of the CAQ is adequate.

### 2.7. Data Analysis

Independent samples *t*-tests were used to calculate baseline differences between treatment completers and noncompleters. Survey data on treatment expectation, program acceptability, and treatment satisfaction were presented descriptively. Participants were regarded to have completed the program only if they had completed all 6 online sessions, allowing a minimal exposure to the six core processes in ACT. Outcome and process variables were analysed using the intention to treat (ITT) principle. Multiple imputation analysis on SPSS IBM Statistics 21 package was conducted. There was one missing value on the SWLS at baseline. The total missing data at posttreatment and follow-up was 9.1%. These missing values were imputed. Paired samples *t*-tests were used to analyze differences at the three assessment time points and Cohen's *d* [45] was used to calculate effect sizes between these assessment time points. A pooled SD was used in these calculations.

IMMPACT recommendations including the convention of using 1/2 SD to calculate clinically meaningful change was followed [46]. The proportion of participants showing clinically meaningful change in the clinical direction was then calculated.

## 3. Results

**Figure 1:**
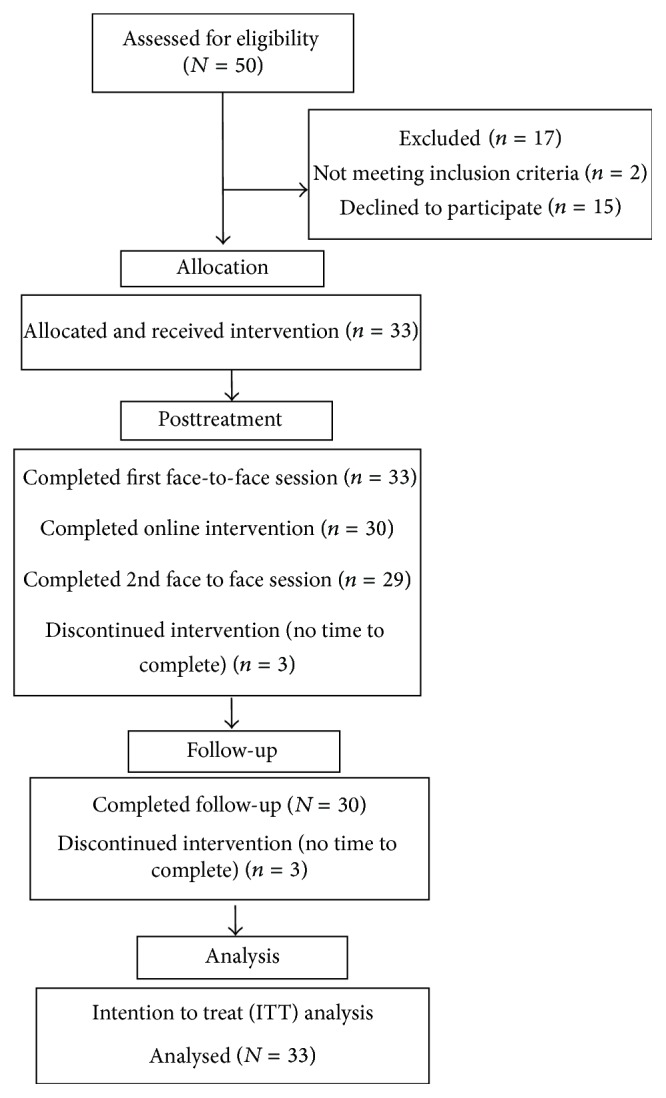
Study flow diagram.

Figure 1 shows the flow of the study.

A total of 64.6% participants who were recruited via the PMC took up treatment. Participants who declined participation cited a lack of interest and time commitments as reasons. Treatment uptake rates for recruitment via the PMC website are not reported as there were limited means to track the total number of people that assessed the website. A total of 90.9% of participants who provided informed consent completed the intervention and provided follow-up data. Out of those who completed the online intervention, only a small percentage (24.2%) accessed the five optional mindfulness-based audio exercises.

A majority of participants (78.8%) were suffering from primary low back pain. A total of 81.8% of participants were seeking specialist treatment, 63.6% were on medication, and 69.7% had undergone physiotherapy. Out of those taking medication, a majority (30.3%) were on anarex, 27.3% were on gabapentin, and 27.2% were prescribed ketoprofen plaster as part of their pain management regime. A smaller percentage (18.2%) of participants were prescribed pregabalin (Lyrica) and nortriptyline. None of the participants on the program were taking strong opioids during program participation.  Table 3 summarises participants' demographics and healthcare usage.

Treatment completers (*n* = 30) and noncompleters (*n* = 3) did not differ on demographic variables and healthcare usage at baseline. However noncompleters demonstrated a significantly higher impact of depression, *t*(31) = 2.14, *p* = 0.04, and lower pain acceptance, *t*(31) = −2.52, *p* = 0.02.

### 3.1. Treatment Expectations, Program Acceptability, and Satisfaction

Participants had expected a reduction of 60.3% in limitations due to pain as a result of program participation, but only a 44.7% reduction in limitations at posttreatment was reported. A reduction of 30.2% in limitations due to pain was maintained at follow-up.  Table 4 summarises participants' treatment expectations.

On measures of program acceptability, responses of “agree” and “strongly agree” were combined to represent “agree” while responses of “disagree” and “strongly disagree” were combined to represent “disagree.” Table 5 summarises the responses of participants on program acceptability and treatment satisfaction. Overall program was acceptable to the majority of participants; 81.8% of participants were generally satisfied with overall treatment. 51.5% continued to access the program and 75.8% continued to practice the strategies at follow-up.

An average of 15.64 (SD = 11.20, range = 8–57) e-mail correspondences transpired between the therapist and each participant during the course of the online program. Total correspondences included a minimum of eight e-mails initiated from the therapist, typically at the start of each session, at program completion, and at follow-up. A majority of participants (63.6%) kept the e-mail correspondences with the therapist to a maximum of 11 e-mails during the course of the program. E-mail correspondences initiated by participants primarily focused on discussions regarding (1) program content and its applications (*M* = 4.76, SD = 7.64, and range = 0–34), (2) engagement and completion of online exercises (*M* = 1.16, SD = 2.16, and range = 0–9), and (3) technical support (*M* = 1.18, SD = 2.47, and range = 0–12); in particular, program navigation and ensuring that data input on the program were successfully saved for future reference. All participant initiated e-mails were responded to within 24 hours of receipt. A frequency of 0 to 3 e-mail correspondences per participant was recorded for each 24-hour time period.

Calls received by the therapist from 30% of participants included a mix of technical related issues and simple clarifications of general issues pertaining to the program. As already mentioned, the added phone call communication made available to participants was not designed to be used as an alternative mode for therapeutic intervention. The phone calls were specifically meant for use when technical support was needed and hence not expected to contain therapeutic content. As such, neither the call duration nor the frequency of calls was specifically recorded, only the general content of each call. None of the calls required any extra therapeutic intervention in addition to the program itself.

### 3.2. Outcomes and Effect Sizes

Table 6 summarises the means (*M*) and standard deviations (SD) obtained at the three assessment time points for all outcomes and PF. Significant improvements in depression at posttreatment, *t* = 3.08, *p* = 0.002, and follow-up, *t* = 3.28, *p* = 0.001, and for pain intensity at follow-up, *t* = 2.15, *p* = 0.03, were demonstrated. All other outcomes were not significant.

Minimal to small effect sizes (*d* = 0.14 to 0.35) were obtained for all outcomes except for a medium effect size for depression (*d* = 0.51). Minimal effect sizes (*d* = 0.02 to 0.09) were obtained for all PF measures.  Table 7 summarises the mean differences and effect sizes at the three assessment time points.

### 3.3. Clinically Meaningful Change

Meaningful change outcomes were generally consistent from posttreatment to follow-up; therefore only follow-up results are reported. Clinically meaningful improvement in at least one outcome (out of five total) was demonstrated in 75.8% of participants; 57.6% made clinically meaningful improvements on at least 2 outcomes, 30.3% on at least 3 outcomes, 18.2% on at least 4 outcomes, and 3.0% on all 5 outcomes. Of those that did not report meaningful improvement, a significant proportion showed no change, 36.4% (satisfaction with life and pain intensity) to 57.6% (impact of depression). A small proportion of participants reported meaningful decline, predominantly a decline in satisfaction with life (24.2%).  Table 8 shows the proportions of participants who meaningfully improved, showed no change, and declined.

## 4. Discussion

Successful recruitment, low drop-out rates, high ratings of overall program acceptability and satisfaction, and significant small effects on depression and pain intensity at 3-month follow-up support the potential feasibility of an ACT-based, combined face-to-face and internet-delivered treatment for people with chronic pain in Singapore.

Results demonstrated that a moderately high percentage of participants (66.7%) had their treatment expectations met. This possibly implied that pretreatment expectations of this study sample matched the purpose of the program. Pretreatment expectations have been shown to predict treatment outcome of CBT interventions in a group of chronic pain patients [47]. The size of our study did not allow for such analyses.

In general, studies have shown that treatment programs aimed at lifestyle changes and including a focus on behavior modification can reach an average nonadherence rate of up to 40% [48, 49]. As high as a 60% nonadherence rate has also been recorded in some studies [12]. Comparatively, the rate of nonadherence shown on this study was small, and this result deserves some discussion. Success with treatment adherence is dependent on a set of key factors [50] and some of these have been provided for in this study. These include (a) a thorough assessment of patients' understanding of the proposed treatment, (b) open communication with the therapist, and (c) an empathic relationship and mutual collaboration between the therapist and the patient. Potentially, other factors such as treatment beliefs, positive attitudes towards treatment, good social support, and a cultural connection with the therapist [50] may have also contributed to the level of adherence and also treatment satisfaction observed here. These factors warrant further exploration.

In reviews of internet-based trials, it is apparent that higher dropout rates coincide with trials that include the lowest level of therapist contact [12, 51]. The added therapist contact time with the inclusion of face-to-face sessions could have contributed to lower dropout rates in this study. The assurance of a quick response from the therapist may have further contributed to the positive effects observed. In addition, other factors such as (a) the varied use of audio-visual tools on the program, (b) participants being able to complete “homework” assignments at their own pace, (c) reduced inconvenience of scheduling multiple appointments with the psychologist, (d) reduced travelling and waiting time to see the psychologist, and (e) reduced stigma associated with seeing a psychologist could all have contributed to participants' motivations and enhanced levels of treatment adherence [52]. Overall, this low attrition rate indicates good feasibility for a future larger scale study.

Unlike previous studies [24, 27], a significant increase in pain acceptance was not found in our sample. Based on the current results, the intervention was associated with a decrease in depression. It has already been demonstrated that internet-based ACT can reduce depression [27, 53] and our results here add to previous findings. The study design here however limits the conclusion of a causal relationship.

It was interesting to observe significant pain reduction and depression in this sample although this was not a primary focus of treatment. When this happens in ACT treatment, it is likely the result of a process in which chronic pain sufferers continue to engage in meaningful activities struggles less to control pain such that the impacts of pain and distress are significantly reduced over time [42, 54]. We note however that the small sample size here limited our power to detect effects and to test potential mediators.

Overall, results demonstrating clinically meaningful improvement across the treatment outcomes at follow-up are encouraging, 27.3% (impact of depression) to 45.5% (pain intensity and depression). A proportion of participants, for example, 6% (depression) to 24.2% (satisfaction with life) reported clinically meaningful decline at follow-up (in some cases these rates were higher immediately after treatment). It is possible that (a) these participants were experiencing natural flare-ups in symptoms as a part of healthy engagement, (b) they may have become more aware of their difficulties or more willing to report them, or (c) perhaps there were some unexpected adverse effects included in the treatment experience. Perhaps, those that declined did not respond as well to online treatment delivery and needed more intensive treatment for positive change to occur. Exploring these speculations, perhaps qualitatively, may contribute further understanding of this result.

Results did not support convincing improvements in pain interference nor satisfaction with life at any of the assessment time points. Nonsignificant findings with minimal effect sizes were also found for all measures of PF. PF has been shown to be relevant for a chronic pain population in Singapore, with PF contributing significant variance to pain interference, depression, and impact of depression beyond pain intensity [34]. Hence, this could mean that (a) the treatment content intended to target these variables may need to be delivered with higher intensity for change to occur, (b) other processes within PF could have shifted in treatment but these were not assessed, (c) the study lacked power to detect significant changes in these domains, or (d) perhaps there were some aspects of the population that were not taken into account in delivery. As our online delivery platform was a first generation prototype, some additional treatment development may be needed and perhaps a better powered study to further explore these speculations.

Like many studies focused on measuring PF, measures of PF here included only selected individual facets of PF. A common objection is that measuring PF using separate measures does not guarantee that these measures are actually measuring precise and independent PF processes [55]. Furthermore, studies measuring PF have typically adopted linear regression models in group data to demonstrate a relationship between individual measures of PF and functioning [55]. So, although strong relationships between PF and physical and emotional functioning have been consistently demonstrated, components of PF overlap and are expected to correlate and share variance. A reliance on regression analyses alone may no longer be sufficient to make a strong case for validating measures of PF [55]. Findings from a recent study [56], focused on measures of PF for people with chronic pain, indicated that a single general factor of PF may exist. This general factor appeared to mainly reflect PF components of “openness,” “decentering,” and “committed action.” As the study was only preliminary, the most effective way to measure these components of PF still require further study at this point. More effective means of assessment and perhaps different study designs will be needed in the future to improve upon current results.

Optimal design of internet-based treatments for chronic pain is essential if it has to produce behaviour change outcomes that are similar to face-to-face treatments. This design will need to include optimal impact on components of PF on outcome via features of the iACT-CEL program. This warrants considerations, such as (a) the choice of metaphors and experiential exercises to include, (b) the optimal number of metaphors and experiential exercises and how to distribute them over time, (c) the number and length of each session, and (d) associated processes such as rapport and therapeutic alliance, may add further utility to future treatment design. Perhaps, a focus on developing more effective adaptions of culturally sensitive elements in the delivery of ACT-based treatment within the context of the intervention can be applied. Such elements may not have been designed and delivered optimally here. A more rigorous inclusion of (a) cultural appropriateness of language, (b) concordance between the therapist and patient, (c) commonly understood concepts within the cultural group, and (d) specific knowledge of cultural uniqueness in treatment content [57] may contribute to better treatment outcome. Recruiting participants with more severe pain, disability, and distress and including a longer follow-up period of 6 months should also be considered for future studies.

Treatments that use computer and communications technology have the potential to reduce treatment costs [10]. The cost effectiveness of the iACT-CEL program was however not measured in this study. The small sample size and the study design included here did not allow for a comprehensive cost-effective and cost-utility analysis. Demonstrating that an internet-delivered treatment is cost-effective can potentially influence policy makers to direct healthcare resources toward developing these. Treatment programs optimally designed and demonstrated to reduce healthcare costs and reduce access barriers can further contribute to optimal care and service delivery for patients with chronic pain.

### 4.1. Study Limitations

This study has a few limitations. Firstly, the study design did not allow for observed changes in outcomes to be attributed to the ACT-based treatment itself. The choice of an uncontrolled study design for the current study seemed most appropriate at this point for a feasibility trial of a never tested culturally adapted treatment with so many unknown elements.

Secondly, the sample size was small. A sample size of minimum *N* = 30 has been recognized as a reasonable minimum sample size needed for parameter estimates of a larger RCT [58, 59]. So although the sample size of this study meets this minimum criterion set for a feasibility study, the small sample size also implies limited statistical power and potentially limited reliability.

Participants were predominantly recruited from one specialist pain treatment clinic. Examining the reliability and generalizability of the present findings with a different and larger sample, including a longer follow-up period may be needed.

Treatment content of the iACT-CEL program was intended to be culturally sensitive and adapted to the Singapore chronic pain population. However, it is difficult to assess whether the adapted aspects were optimal; this would require some comparison between differing versions of treatment. Treatment-related competency and fidelity were also not formally assessed. The challenges of treatment optimisation and integrity remain a priority for future studies.

## 5. Conclusions

The current study of an ACT-based treatment, examined in the healthcare context of Singapore, showed that it appears feasible and potentially promising for future research and development. Future studies will need to consider more effective ways to target outcomes of pain interference, satisfaction with life and processes related to PF, which contrary to expectations did not demonstrate a convincing pattern of significant change here. There are features to consider in the future, such as number of sessions to include, frequency in the delivery of treatment content, choice of delivery modes, and tracking for time spent in treatment. Features that may potentially influence treatment outcome.

## Figures and Tables

**Table 1:** Schematic overview of iACT-CEL intervention.

Sessions	Information	Assignments	Audio/video
1st face-to-face session prior to online session	Introduction to iACT-CEL. Experiential acceptance exercise: Chinese Finger Trap	Goal-setting exercise	—
*Online sessions*			
Session 1			
The problem with avoidance	Pain avoidance cycle	Evaluation of coping and avoidance strategies	Tug of War (animation)
	Acceptance	Noticing feelings and occasions of struggling	Joe the Bum (animation)
Session 2			
More on openness and you are not your thoughts	Concept of cognitive fusion & defusion introduced	Working on fused thoughts	Passengers on the bus (animation)
			Expansion exercise
Session 3			
I accept	Concept of present moment awareness introduced	Reflection on current thoughts and emotions	I'm having the thought that (video)
Session 4			
In the present moment	Concept of self as context introduced	Reflection on the observed self	Notice 5 things (video)
			The observing self (audio)
Session 5			
What do you want out of life?	Concept of values introduced	Values clarification	Get off your “buts” (video)
		Reflection of values	80th Birthday (animation)
Session 6			
Committed action	Concept of committed action introduced	Willingness and action plan	The Swamp (video)
2nd face-to-face session after 6th online session	Committed action	Goal-setting exercise	—

**Table 2:** Summary of Study Schedule.

Week 0	Week 1	Week 2	Week 3	Week 4	Week 5 + 3-month follow-up
Receive study information and provide informed consent. Receive a unique username and password to log on to the online program and to a separate secure e-mail account created for the purposes of this study. Receive an e-mail link to complete a set of baseline questionnaires online. A first face-to-face session with the therapist is scheduled via e-mail and a follow-up phone call.	Complete the first face-to-face session with the therapist. Learn to navigate through the online program and receive instructions regarding participant-therapist communication on the program. Receive an e-mail link to the welcome page of the online program (http://www.iactcel.com).	Complete sessions 1 and 2 on the online program. Complete a set of diary ratings on a scale of 0–10 rating their level of struggling versus openness to pain at the end of week 2–4. Ratings used for treatment purposes only. Access to the next session is given upon satisfactory completion of the previous session. Access to 5 optional online audio exercises.	Complete sessions 3 and 4 on the online program.	Complete sessions 5 and 6 on the online program. Contacted via e-mail to schedule the final face-to-face session with the therapist.	Complete the final face to face session with the therapist. Receive an e-mail link to complete a set of posttreatment questionnaires online at week 5 and a similar set of follow-up questionnaires at 3-month follow-up.

**Table 3:** Summary of participants' demographics.

	Mean (SD)
Age	47.61 (12.63)
Years of education	13.61 (2.93)
Pain duration	111.39 (91.79)
Medical leave days	21.64 (66.76)
Number of doctors seen	3.00 (2.13)
Number of doctor visits	2.39 (2.05)
Number of A & E visits	0.00 (0.00)

	Participants number (%)

Sex	
Male	8 (24.2)
Female	25 (75.8)
Race	
Chinese	22 (66.7)
Malay	4 (12.1)
Indian	2 (6.1)
Others	5 (15.2)
Marital	
Married	11 (33.3)
Divorced	4 (12.1)
Single	18 (54.5)
Housing	
Lives with spouse and children	11 (33.3)
Lives with parents	13 (39.4)
Lives alone	3 (9.1)
Other	2 (6.1)
Work Status	
Full time	18 (54.5)
Part time	5 (15.2)
Others	10 (30.4)

**Table 4:** Summary of participants' pre- and posttreatment expectations.

(*N* = 33)	Not at All (%)	A little (%)	Reasonably (%)	Strongly (%)	Very strongly (%)
Program can help to manage pain	0 (0.0)	7 (21.2)	15 (45.5)	9 (27.3)	2 (6.1)

(*N* = 33)	Extremely unsuccessful (%)	Unsuccessful (%)	Neutral (%)	Successful (%)	Extremely successful (%)

Expectation of success at reducing limitations due to pain	0 (0.0)	1 (3.0)	10 (30.3)	20 (60.6)	2 (6.1)

	Highly unmet (%)	Unmet (%)	Neither met nor unmet (%)	Met (%)	Highly met (%)

Posttreatment expectations	0 (0.0)	1 (3.3)	7 (21.2)	18 (54.5)	4 (12.1)

	Extremely unsuccessful (%)	Unsuccessful (%)	Neutral (%)	Successful (%)	Extremely successful (%)

Program success in reducing limitations due to pain	0 (0.0)	3 (9.1)	13 (39.4)	14 (42.4)	0 (0.0)

**Table 5:** Summary of participants' responses on program acceptability and treatment satisfaction.

(*N* = 33)		Disagree (%)	Neither agree nor disagree (%)		Agree (%)
Information on the program was easy to understand		1 (3.0)	6 (18.2)		23 (69.7)
Information was personally relevant		1 (3.0)	3 (9.1)		26 (78.8)
Program was easy to use		1 (3.0)	4 (12.1)		25 (75.8)
Interactive exercises were helpful		1 (3.0)	4 (12.1)		25 (75.7)
Ability to communicate to the therapist via e-mail was important		0 (0.0)	14 (42.4)		16 (48.5)
Ability to apply techniques learnt in daily life		1 (3.0)	8 (24.2)		21 (63.6)
No technical difficulties were experienced		7 (21.2)	3 (9.1)		20 (60.6)
Duration of program was just right		3 (9.1)	1 (3.0)		26 (78.8)
Program likely to help people with chronic pain manage more effectively		1 (3.0)	6 (18.2)		23 (69.7)

Treatment satisfaction	Extremely unsatisfied (%)	Unsatisfied (%)	Neutral (%)	Satisfied (%)	Extremely satisfied (%)

Therapist's response time	0 (0.0)	0 (0.0)	7 (21.2)	12 (36.4)	11 (33.3)
Quality of interaction with therapist	0 (0.0)	0 (0.0)	3 (9.1)	18 (54.5)	9 (27.3)
Online program	0 (0.0)	0 (0.0)	6 (18.2)	17 (51.5)	7 (21.2)
Total treatment (including face-to-face sessions)	0 (0.0)	0 (0.0)	3 (9.1)	18 (54.5)	9 (27.3)

**Table 6:** Summary of means and standard deviations for outcomes and process measures.

(*N* = 33)	Baseline mean (SD)	Posttreatment mean (SD)	Follow-up mean (SD)
Pain interference	4.50 (2.81)	3.90 (2.60)	3.91 (2.37)
Satisfaction with life	18.41 (8.25)	20.30 (8.85)	19.18 (10.33)
Pain intensity	5.29 (2.70)	4.90 (2.72)	4.37 (2.37)
Depression	11.15 (6.31)	8.14 (5.54)	8.86 (5.91)
Impact of depression	0.91 (0.67)	0.67 (0.67)	0.79 (0.58)
Pain acceptance	25.21 (6.93)	25.92 (9.53)	25.44 (9.60)
General acceptance	26.36 (9.90)	26.13 (10.91)	25.68 (13.83)
Committed action	65.73 (17.00)	66.82 (17.47)	64.95 (19.63)

**Table 7:** Mean differences and effect sizes for baseline to posttreatment and baseline to follow-up.

(*N* = 33)	Baseline to posttreatment (*t*-test)	Cohen's *d*	Baseline to follow-up (*t*-test)	Cohen's *d*
Pain interference	*t* = 1.17, *p* = 0.24	0.22	*t* = 1.59, *p* = 0.11	0.22
Satisfaction with life	*t* = −0.70, *p* = 0.49	0.22	*t* = −0.27, *p* = 0.79	0.09
Pain intensity	*t* = 0.90, *p* = 0.37	0.14	*t* = 2.15, *p* = 0.03	0.34
Depression	*t* = 3.08, *p* = 0.002	0.51	*t* = 3.28, *p* = 0.001	0.39
Impact of depression	*t* = 1.95, *p* = 0.06	0.35	*t* = 0.98, *p* = 0.33	0.18
Pain acceptance	*t* = −0.30, *p* = 0.77	0.09	*t* = −0.10, *p* = 0.92	0.03
General acceptance	*t* = 0.17, *p* = 0.87	0.02	*t* = 0.29, *p* = 0.78	0.07
Committed action	*t* = −0.34, *p* = 0.73	0.06	*t* = 0.14, *p* = 0.89	0.05

**Table 8:** Proportions of participants who made clinically meaningful improvements showed no change and declined.

(*N* = 33)	Improved (%)		No change (%)		Declined (%)	
	Posttreatment	F/U	Posttreatment	F/U	Posttreatment	F/U
Pain interference	11 (33.3)	10 (30.3)	17 (51.5)	17 (51.5)	5 (15.2)	6 (18.2)
Satisfaction with life	13 (39.4)	13 (39.4)	11 (33.3)	12 (36.4)	9 (27.3)	8 (24.2)
Pain intensity	14 (42.4)	15 (45.5)	7 (21.0)	12 (36.4)	12 (36.4)	6 (18.2)
Depression	12 (36.4)	15 (45.5)	18 (54.5)	16 (48.5)	3 (9.0)	2 (6.0)
Impact of depression	8 (24.3)	9 (27.3)	24 (72.7)	19 (57.6)	1 (3.0)	5 (15.2)

F/U: follow-up.

## Appendix

### A. Items Measuring Pre- and Posttreatment Expectations

#### A.1. Pretreatment Expectations

1. Do you expect that this treatment program will help you to manage your pain better?
    1: Not at all
    2: A little
    3: Reasonably
    4: Strongly
    5: Very strongly
2. How successful do you think this treatment will be in reducing your limitations due to pain?
    1: Extremely unsuccessful
    2: Unsuccessful
    3: Neither successful nor unsuccessful
    4: Successful
    5: Extremely successful
3. By the end of treatment, how much improvements in your limitations due to pain do you feel will occur?
    0%
    10%
    20%
    30%
    40%
    50%
    60%
    70%
    80%
    90%
    100%

#### A.2. Posttreatment Expectations

1. Were your expectations of the treatment program met?
    1: Highly unmet
    2: Unmet
    3: Neither met nor unmet
    4: Met
    5: Highly met
2. How successful was the treatment in reducing your limitations due to pain?
    1: Extremely unsuccessful
    2: Unsuccessful
    3: Neither successful nor unsuccessful
    4: Successful
    5: Extremely successful
3. How much improvements in your limitations due to pain have occurred?
    0%
    10%
    20%
    30%
    40%
    50%
    60%
    70%
    80%
    90%
    100%

### B. Survey on Program Acceptability and Treatment Satisfaction

1. We would like your opinion on the following aspects of the iACT-CEL program. Please rate your answers on a scale of 1 to 5 where “1” represents “strongly disagree” and “5” represents “strongly agree”.
  - I found the information on the program easy to understand
      1: Strongly disagree
      2: Disagree
      3: Neither agree nor disagree
      4: Agree
      5: Strongly agree
  - I found the information to be personally relevant
      1: Strongly disagree
      2: Disagree
      3: Neither agree nor disagree
      4: Agree
      5: Strongly agree
  - I found the program easy to use
      1: Strongly disagree
      2: Disagree
      3: Neither agree nor disagree
      4: Agree
      5: Strongly agree
  - I found the interactive exercises helpful
      1: Strongly disagree
      2: Disagree
      3: Neither agree nor disagree
      4: Agree
      5: Strongly agree
  - The ability to communicate with the therapist via e-mail was important to me
      1: Strongly disagree
      2: Disagree
      3: Neither agree nor disagree
      4: Agree
      5: Strongly agree
  - I am able to apply the techniques learnt on the program in my daily life
      1: Strongly disagree
      2: Disagree
      3: Neither agree nor disagree
      4: Agree
      5: Strongly agree
  - The duration of the program was just right
      1: Strongly disagree
      2: Disagree
      3: Neither agree nor disagree
      4: Agree
      5: Strongly agree
  - The iACT-CEL program is likely to help people with chronic pain manage pain more effectively.
      1: Strongly disagree
      2: Disagree
      3: Neither agree nor disagree
      4: Agree
      5: Strongly agree
2. How satisfied were you with the iACT-CEL program? Please rate your satisfaction of each item on a scale of 1 to 5 where “1” represents “extremely unsatisfied” and “5” represents “extremely satisfied”.
  - How satisfied are you with the response time of the therapist on the program?
      1: Extremely unsatisfied
      2: Unsatisfied
      3: Neither satisfied nor unsatisfied
      4: Satisfied
      5: Extremely satisfied
  - How satisfied are you with the quality of the interaction with the therapist on the program?
      1: Extremely unsatisfied
      2: Unsatisfied
      3: Neither satisfied nor unsatisfied
      4: Satisfied
      5: Extremely satisfied
  - How satisfied are you with the iACT-CEL online program?
      1: Extremely unsatisfied
      2: Unsatisfied
      3: Neither satisfied nor unsatisfied
      4: Satisfied
      5: Extremely satisfied
  - How satisfied are you with the total treatment (including the face to face sessions)?
      1: Extremely unsatisfied
      2: Unsatisfied
      3: Neither satisfied nor unsatisfied
      4: Satisfied
      5: Extremely satisfied

## References

[1] Breivik H., Collett B., Ventafridda V., Cohen R., Gallacher D. (2006). Survey of chronic pain in Europe: prevalence, impact on daily life, and treatment. *European Journal of Pain*.

[2] Johannes C. B., Le T. K., Zhou X., Johnston J. A., Dworkin R. H. (2010). The prevalence of chronic pain in united states adults: results of an internet-based survey. *Journal of Pain*.

[3] Nakamura M., Nishiwaki Y., Ushida T., Toyama Y. (2014). Prevalence and characteristics of chronic musculoskeletal pain in Japan: a second survey of people with or without chronic pain. *Journal of Orthopaedic Science*.

[4] Drayer R. A., Henderson J., Reidenberg M. (1999). Barriers to better pain control in hospitalized patients. *Journal of Pain and Symptom Management*.

[5] Fishbain D., Cutler R. B., Rosomoff H. L., Rosomoff R. S. (2000). What is the quality of the implemented meta-analytic procedures in chronic pain treatment meta-analyses?. *Clinical Journal of Pain*.

[6] Fullen B. M., Doody C., David Baxter G., Daly L. E., Hurley D. A. (2008). Chronic low back pain: non-clinical factors impacting on management by Irish doctors. *Irish Journal of Medical Science*.

[7] Yang S.-Y., Bogosian A., Moss-Morris R., Mccracken L. M. (2015). Mixed experiences and perceptions of psychological treatment for chronic pain in singapore: skepticism, ambivalence, satisfaction, and potential. *Pain Medicine*.

[8] Glajchen M. (2001). Chronic pain: treatment barriers and strategies for clinical practice. *Journal of the American Board of Family Practice*.

[9] Lohman D., Schleifer R., Amon J. J. (2010). Access to pain treatment as a human right. *BMC Medicine*.

[10] Griffiths F., Lindenmeyer A., Powell J., Lowe P., Thorogood M. (2006). Why are health care interventions delivered over the internet? A systematic review of the published literature. *Journal of Medical Internet Research*.

[11] Keogh E., Rosser B. A., Eccleston C. (2010). E-Health and chronic pain management: current status and developments. *Pain*.

[12] Cuijpers P., Van Straten A., Andersson G. (2008). Internet-administered cognitive behavior therapy for health problems: a systematic review. *Journal of Behavioral Medicine*.

[13] Flor H., Turk D. C. (2011). *Chronic Pain: An Integrated Biobehavioral Approach*.

[14] Turk D. C. (2003). Cognitive-behavioral approach to the treatment of chronic pain patients. *Regional Anesthesia and Pain Medicine*.

[15] McCracken L. M., Vowles K. E. (2014). Acceptance and commitment therapy and mindfulness for chronic pain: model, process, and progress. *American Psychologist*.

[16] Yang S.-Y., McCracken L. M. (2014). Acceptance and commitment therapy for chronic pain. *Journal of Clinical Outcomes Management*.

[17] Hayes S. C., Strosahl K. D., Wilson K. G. (1999). *Acceptance and Commitment Therapy: An experiential approach to behavior change*.

[18] Hayes S. C., Strosahl K. D., Wilson K. G. (2012). *Acceptance and Commitment Therapy: The Process and Practice of Mindful Change*.

[19] Scott W., McCracken L. M. (2015). Psychological flexibility, acceptance and commitment therapy, and chronic pain. *Current Opinion in Psychology*.

[20] McCracken L. M., Gutiérrez-Martínez O. (2011). Processes of change in psychological flexibility in an interdisciplinary group-based treatment for chronic pain based on Acceptance and Commitment Therapy. *Behaviour Research and Therapy*.

[21] Vowles K. E., Witkiewitz K., Sowden G., Ashworth J. (2014). Acceptance and commitment therapy for chronic pain: evidence of mediation and clinically significant change following an abbreviated interdisciplinary program of rehabilitation. *Journal of Pain*.

[22] Wicksell R. K., Olsson G. L., Hayes S. C. (2010). Psychological flexibility as a mediator of improvement in Acceptance and Commitment Therapy for patients with chronic pain following whiplash. *European Journal of Pain*.

[23] McCracken L. M., Gutieŕrez-Martínez O., Smyth C. (2013). ‘Decentering’ reflects psychological flexibility in people with chronic pain and correlates with their quality of functioning. *Health Psychology*.

[24] McCracken L. M. (2013). Committed action: an application of the psychological flexibility model to activity patterns in chronic pain. *Journal of Pain*.

[25] Voss Horrell S. C. (2008). Effectiveness of cognitive-behavioral therapy with adult ethnic minority clients: a review. *Professional Psychology: Research and Practice*.

[26] La Roche M. (2012). *Cultural Psychotherapy: Theory, Methods and Practice, Sage*.

[27] Buhrman M., Skoglund A., Husell J. (2013). Guided internet-delivered acceptance and commitment therapy for chronic pain patients: a randomized controlled trial. *Behaviour Research and Therapy*.

[28] Trompetter H. R., Bohlmeijer E. T., Veehof M. M., Schreurs K. M. G. (2014). Internet-based guided self-help intervention for chronic pain based on Acceptance and Commitment Therapy: a randomized controlled trial. *Journal of Behavioral Medicine*.

[29] Hann K. E. J., McCracken L. M. (2014). A systematic review of randomized controlled trials of Acceptance and Commitment Therapy for adults with chronic pain: outcome domains, design quality, and efficacy. *Journal of Contextual Behavioral Science*.

[30] Yang S.-Y., Bogosian A., Moss-Morris R., McCracken L. M. (2016). Healthcare professionals’ perceptions of psychological treatment for chronic pain in Singapore: challenges, barriers, and the way forward. *Disability and Rehabilitation*.

[31] Infocomm Development Authority of Singapore (IDA) http://www.ida.gov.sg/Tech-Scene-News/Facts-and-Figures.

[32] Yang S. Y. (2016). *Development of a Psychologically-Based Treatment for Chronic Pain in Singapore: Patient and Healthcare Professional Inputs, Theoretical Model and a Feasibility Trial*.

[33] Eccleston C., Fisher E., Craig L., Duggan G. B., Rosser B. A., Keogh E. (2014). Psychological therapies (internet-delivered) for the management of chronic pain in adults. *The Cochrane Database of Systematic Reviews*.

[34] Yang S., McCracken L. M., Moss-Morris R. (2016). Psychological treatment needs for chronic pain in singapore and the relevance of the psychological flexibility model. *Pain Medicine*.

[35] Borkovec T. D., Nau S. D. (1972). Credibility of analogue therapy rationales. *Journal of Behavior Therapy and Experimental Psychiatry*.

[36] Cleeland C. S., Ryan K. M. (1994). Pain assessment: global use of the Brief Pain Inventory. *Annals of the Academy of Medicine Singapore*.

[37] Keller S., Bann C. M., Dodd S. L., Schein J., Mendoza T. R., Cleeland C. S. (2004). Validity of the brief pain inventory for use in documenting the outcomes of patients with noncancer pain. *Clinical Journal of Pain*.

[38] Diener E., Emmons R. A., Larsem R. J., Griffin S. (1985). The satisfaction with life scale. *Journal of Personality Assessment*.

[39] Von Korff M., Ormel J., Keefe F. J., Dworkin S. F. (1992). Grading the severity of chronic pain. *Pain*.

[40] Kroenke K., Spitzer R. L., Williams J. B. W. (2001). The PHQ-9: validity of a brief depression severity measure. *Journal of General Internal Medicine*.

[41] Fish R. A., McGuire B., Hogan M., Morrison T. G., Stewart I. (2010). Validation of the Chronic Pain Acceptance Questionnaire (CPAQ) in an Internet sample and development and preliminary validation of the CPAQ-8. *Pain*.

[42] McCracken L. M., Vowles K. E., Eccleston C. (2004). Accepfctance of chronic pain: component analysis and a revised assessment method. *Pain*.

[43] Bond F. W., Hayes S. C., Baer R. A. (2011). Preliminary psychometric properties of the acceptance and action questionnaire-II: a revised measure of psychological inflexibility and experiential avoidance. *Behavior Therapy*.

[44] Hayes S. C., Strosahl K., Wilson K. G. (2004). Measuring experiential avoidance: a preliminary test of a working model. *Psychological Record*.

[45] Cohen J. (1988). *Statistical Power Analysis for the Behavioral Sciences*.

[46] Dworkin R. H., Turk D. C., Wyrwich K. W. (2008). Interpreting the clinical importance of treatment outcomes in chronic pain clinical trials: immpact recommendations. *Journal of Pain*.

[47] Goossens M. E. J. B., Vlaeyen J. W. S., Hidding A., Kole-Snijders A., Evers S. M. A. A. (2005). Treatment expectancy affects the outcome of cognitive-behavioral interventions in chronic pain. *Clinical Journal of Pain*.

[48] DiMatteo M. R., Nicola D. D. (1982). *Achieving Patient Compliance*.

[49] Haynes R. B., McDonald H., Garg A. X., Montague P. (2002). Interventions for helping patients to follow prescriptions for medications. *Cochrane database of systematic reviews (Online)*.

[50] Martin L. R., Williams S. L., Haskard K. B., DiMatteo M. R. (2005). The challenge of patient adherence. *Therapeutics and Clinical Risk Management*.

[51] MacEa D. D., Gajos K., Daglia Calil Y. A., Fregni F. (2010). The efficacy of web-based cognitive behavioral interventions for chronic pain: a systematic review and meta-analysis. *Journal of Pain*.

[52] Marks I. M., Cavanagh K., Gega L. (2007). *Maudsley Monographs no. 45. Hands-on Help: Computer Aided Psychotherapy*.

[53] Lappalainen P., Granlund A., Siltanen S. (2014). ACT Internet-based vs face-to-face? A randomized controlled trial of two ways to deliver Acceptance and Commitment Therapy for depressive symptoms: an 18-month follow-up. *Behaviour Research and Therapy*.

[54] McCracken L. M., Spertus I. L., Janeck A. S., Sinclair D., Wetzel F. T. (1999). Behavioral dimensions of adjustment in persons with chronic pain: pain-related anxiety and acceptance. *Pain*.

[55] Vowles K. E., Sowden G., Ashworth J. (2014). A comprehensive examination of the model underlying acceptance and commitment therapy for chronic pain. *Behavior Therapy*.

[56] Scott W., McCracken L. M., Norton S. (2016). A Confirmatory factor analysis of facets of psychological flexibility in a sample of people seeking treatment for chronic pain. *Annals of Behavioral Medicine*.

[57] Bernal G., Bonilla J., Bellido C. (1995). Ecological validity and cultural sensitivity for outcome research: issues for the cultural adaptation and development of psychosocial treatments with Hispanics. *Journal of Abnormal Child Psychology*.

[58] Browne R. H. (1995). On the use of a pilot sample for sample size determination. *Statistics in Medicine*.

[59] Hertzog M. A. (2008). Considerations in determining sample size for pilot studies. *Research in Nursing and Health*.

